# Mitigating osteonecrosis of the jaw (ONJ) through preventive dental care and understanding of risk factors

**DOI:** 10.1038/s41413-020-0088-1

**Published:** 2020-03-11

**Authors:** Jason T. Wan, Douglas M. Sheeley, Martha J. Somerman, Janice S. Lee

**Affiliations:** 1 National Institute of Dental and Craniofacial Research, National Institutes of Health, Bethesda, MD USA; 2 Laboratory for Oral Connective Tissue Biology, National Institute of Arthritis and Musculoskeletal and Skin Diseases, National Institutes of Health, Bethesda, MD USA

**Keywords:** Dental diseases, Bone, Pathogenesis

## Abstract

It is well established that alterations in phosphate metabolism have a profound effect on hard and soft tissues of the oral cavity. The present-day clinical form of osteonecrosis of the jaw (ONJ) was preceded by phosphorus necrosis of the jaw, ca. 1860. The subsequent removal of yellow phosphorus from matches in the early 20th century saw a parallel decline in “phossy jaw” until the early 2000s, when similar reports of unusual jaw bone necrosis began to appear in the literature describing jaw necrosis in patients undergoing chemotherapy and concomitant steroid and bisphosphonate treatment. Today, the potential side effect of ONJ associated with medications that block osteoclast activity (antiresorptive) is well known, though the mechanism remains unclear and the management and outcomes are often unsatisfactory. Much of the existing literature has focused on the continuing concerns of appropriate use of bisphosphonates and other antiresorptive medications, the incomplete or underdeveloped research on ONJ, and the use of drugs with anabolic potential for treatment of osteoporosis. While recognizing that ONJ is a rare occurrence and ONJ-associated medications play an important role in fracture risk reduction in osteoporotic patients, evidence to date suggests that health care providers can lower the risk further by dental evaluations and care prior to initiating antiresorptive therapies and by monitoring dental health during and after treatment. This review describes the current clinical management guidelines for ONJ, the critical role of dental-medical management in mitigating risks, and the current understanding of the effects of predominantly osteoclast-modulating drugs on bone homeostasis.

## Background

Osteonecrosis of the jaw (ONJ) is classically considered a disruption of vascular supply or avascular necrosis with exposure of the jaw bones. It can be caused by radiation, high-dose steroid therapy, and medications that disrupt vascular supply or bone turnover in the jaws.^1,2^ Malignancies of, or metastatic disease to, the jaw can also result in osteonecrosis and subsequent exposure of the jaw bones. In the last 20 years, more drug therapies have been introduced to treat both osteoporosis and malignancies with skeletal-related events by slowing bone turnover through antiresorptive functions, i.e., targeting the osteoclasts with bisphosphonates (BPs) and RANK ligand (RANKL) inhibitors. Some examples of drugs used to treat osteoporosis, osteopenia, genetic disorders of mineralized tissues, and cancer-mediated bone effects include alendronate (ALN), zoledronate (ZOL), and denosumab (DNB) (a RANKL inhibitor).^3,4^ Table 1 lists pharmaceutical agents including trade names associated with medication-related ONJ (MRONJ) and their primary use, oncologic or osteoporotic, based on their mode of action. Evidence demonstrates a reduction in risk of vertebral and hip fragility fractures in osteoporotic patients taking such drugs. However, while rare, these agents may cause atypical femur fractures.

**Table 1:** Pharmaceutical agents associated with MRONJ

Pharmaceutical agents associated with MRONJ	Mode of action
**For osteoporosis/bone conditions (trade name)**	
Alendronate (Fosamax)	Nitrogen containing BP inhibits mevalonate pathway
Ibandronate (Boniva)	Nitrogen containing BP inhibits mevalonate pathway
Pamidronate (Aredia)	Nitrogen containing BP inhibits mevalonate pathway
Risedronate (Actonel)	Nitrogen containing BP inhibits mevalonate pathway
Zoledronate (Zometa)	Nitrogen containing BP inhibits mevalonate pathway
Denosumab (Xgeva)	Antibody binds to RANK ligand
Clodronate (Bonefos, Loron)	Nonnitrogen containing BP competes with ATP as metabolite
Etidronate (Didronel)	Nonnitrogen containing BP competes with ATP as metabolite
Tiludronate (Skelid)	Nonnitrogen containing BP competes with ATP as metabolite
**For Oncologic use**	(all these compounds affect angiogenesis)
Imatinib, Sunitinib (Sutent)	Tyrosine kinase inhibitors
Sorafenib (Nexavar)	VEGF inhibitor
Bevacizumab (Avastin)	Angiogenic inhibitor

*BP* Bisphosphonate, *RANK* receptor activator of nuclear factor kappa-Β, *ATP* adenosine triphosphate, *VEGF* vascular endothelial growth factor

In addition, antiangiogenic medications, such as tyrosine kinase inhibitors^5^ or monoclonal antibodies targeting vascular endothelial growth factor (VEGF), have been used as adjuvant therapies for the management of solid tumors and cancer-related conditions such as bone metastases (See Table 1). There is evidence of improvement in quality of life using these therapies to reduce bone pain, but limited evidence to support overall improvement in cancer survival rates. Unfortunately, all these therapeutics are associated with increased risk of MRONJ. It is also well established that BP have antiangiogenic properties^6–9^ and therefore are effective at inhibiting tumor angiogenesis.^10^ Antiangiogenesis drugs affect wound healing and the resulting effects on bone are more pronounced in areas with inherently high bone turnover rates such as in the mandible.^11^ Thus, it is not surprising that antiangiogenic medications are associated with ONJ.

**Fig. 1:**
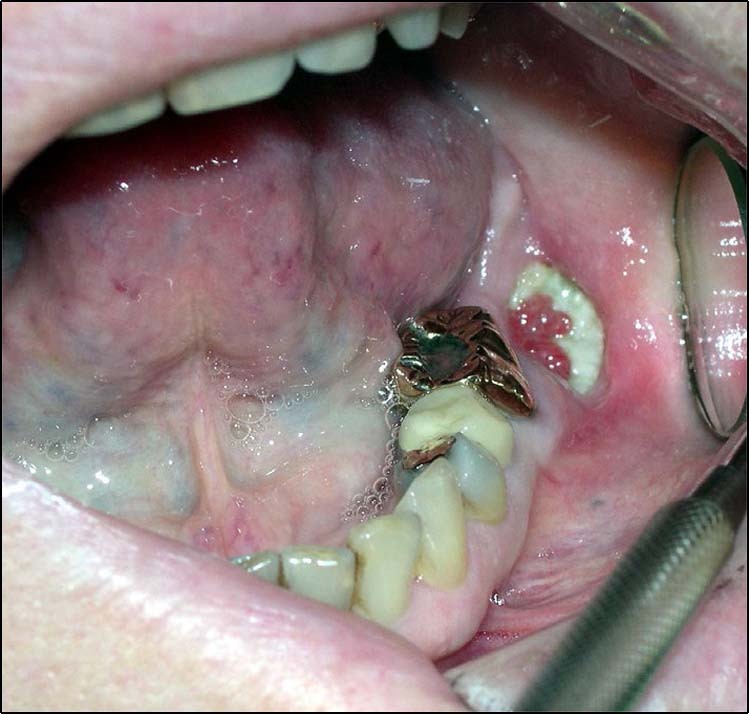
Clinical photo of nonhealing bone after a routine dental extraction with exposure of necrotic bone. 67-year-old female with nonhealing extraction site of the lower left second molar (#18). Patient had a history of metastatic breast cancer and was receiving chemotherapy, prednisone, and zoledronate. Unfortunately, 4 months after the extraction, the site was painful with exposed bone and poor healing consistent with medication-related osteonecrosis of the jaw. Normal bone healing after a dental extraction would have shown mucosal coverage within a month

First-generation BPs, for treatment of osteoporosis, were released in 1995. Oral and maxillofacial surgeons noted and published cases of unusual bone exposure in patients in 2003.^1,12,13^ Dramatic presentation of nonhealing bone after a routine dental extraction, i.e., MRONJ, with exposure of necrotic bone alarmed dental and medical communities and their patients (Fig. 1); though the etiology was slow to be identified as the affected population included osteoporotic patients and patients receiving chemotherapy, high-dose steroids, and BPs.^14,15^ Management included debridement, removal of bone sequestra, jaw resections, control of infections, and subsequent free-tissue composite reconstruction. Unfortunately, the consequences of ONJ, even when infections were controlled, left many individuals debilitated and with chronically exposed bone. The surgical principle of resecting or debriding until bleeding healthy bone is encountered did not apply to these patients as the impact of the BPs was widespread throughout the jaw bones. Interestingly, osteonecrosis appeared to have a distinct predilection for bones of the head and neck region, particularly the mandible (lower jaw) and maxilla (upper jaw).^13,16^

The eerie similarities of bisphosphonate-related ONJ (BRONJ) documented in 2003 to the “phossy jaw”^17^of the late 1800s found in matchmaking factory workers are an unfortunate example of similar environmental and pharmaceutical effects through a common mevalonate pathway that interrupts osteoclast function and slows bone turnover.^18^ “Phossy jaw”, or phosphorus necrosis of the jaw, was an occupational hazard related to the addition of phosphorous to friction match paste. The yellow phosphorous (P_4_O_10_) used in matches to enhance ignition converted to BPs during match manufacturing, which resulted in factory workers exposed to the high concentrations of BP developing necrosis of the lower jaw with unrelenting pain. Two publications this past year, one excellent review by Chang et al. focused on basic mechanisms of ONJ,^19^ and the other, a small study surveying 15 dentists, complement our review focused on the need for team-based treatment for individuals receiving antiresorptive therapy.^20^ The findings that a greater focus on preventive care and discussion with providers at the point of prescribing antiresorptive therapy allow dentists to implement strategies to mitigate risks for developing MRONJ support our conclusions and recommendations for guiding health care providers and their patients, Table 2.

**Table 2:** Guide to clinicians for monitoring patients requiring antiresorptive therapy

**Management**
Overall dental health: • Assess and manage current disease state: teeth present, oral-dental infections, e.g., caries, periodontal disease, and xerostomia (multiple causes) • Assess quality of existing restorations, including dentures, bridges, and implants • Identify and address restorative needs prior to, during, and after therapy and removal of nonsalvageable teeth prior to therapy • Monitor for normal oral hard and soft tissue healing after dental procedures and extractions, and for resolution of oral pain
**Risk factors**
General: • Increasing age • Female gender
Overall systemic conditions that may exacerbate ONJ development: • Compromised immune system • Autoimmune diseases • Diabetes • Mineralized tissue disorders/diseases, especially those known to affect bone homeostasis • Genetic factors with suggested increased risk of ONJ (i.e., specific polymorphism in FDPS gene or SIRT1/HERC4 locus)
Medications that exacerbate ONJ risk: • Concomitant steroids or chemotherapeutic agents • Concomitant antiangiogenic agents
Antiresorptive regimens that increase the risk of ONJ: • Treatment for skeletal-related events in cancer have greater risk than osteoporosis/osteopenia • Intravenous formulations have greater risk than oral forms of bisphosphonates, with risk plateaus at 2–3 years and >4 years, respectively

A famous musical of the late 19th century, “The Matchgirls” by Bill Owen and Tony Russell (1888), was based on this debilitating disease with a song in Act 1, Scene 1 of the musical:

- Top grade selectable
- Hardly detectable
- Phosphorous, phosphorous
- Taste is more subtler and
- Spreads just like butter-grand
- Phosphorous, phosphorous
- Our special beauty cream
- We look a proper dream -
- For we are minus a jaw
- Guv’nors don’t charge a fee
- Give it away for free
- Phosphorous, phosphorous, phosphorous

Credit: The Matchgirls by Bill Owen published by Samuel French Ltd. Reprinted by permission of Samuel French Ltd (A Concord Theatrical Company) and Lemon Una & Durbridge (United Agents LLP).

The risk of ONJ is relatively low.^21^ However, this may be underreported because bone exposure may be a late presentation of ONJ.^22–24^ The consequences of ONJ and the subsequent therapies are costly, time consuming, and can result in significant debilitation of patients.^25,26^ For patients and care providers alike, the most critical factor for limiting the risk of developing MRONJ is through optimizing patients’ oral health prior to initiating therapy. It is essential to incorporate dentists and dental hygienists in the multidisciplinary care of these at-risk patients.^27^ Dental screening and appropriate oral care prior to initiating and during antiresorptive and antiangiogenic therapy lowered the risk of ONJ by 50%.^28–30^ These preventive efforts would be equivalent to preparing a patient before cardiac surgery or before initiating radiation therapy for head and neck cancers. Engaging health care providers to make preventive dental care a routine part of pretreatment management should be a national priority for both overall good clinical practice and cost-effective management.

### Management

National and international dental, medical, and mineralized tissue research and practice organizations have presented their positions on prevention and management of ONJ and have urged all providers to discuss risks and benefits of antiresorptive therapy with patients. They highlight prevention strategies for ONJ that include elimination or stabilization of oral disease prior to initiation of antiresorptive agents, as well as maintenance of good oral hygiene. For patients at high risk for the development of ONJ, consideration should be given to withholding antiresorptive therapy before and following extensive oral surgery until the surgical site heals with mature mucosal coverage.^2,31–34^ Preventive care may include dental prophylaxis, caries control, restorative dental procedures, and removal of nonrestorable teeth. If extractions are needed, existing recommendations state that patients should wait 14–21 days after dental extractions before initiating drug therapy.^2^

**Fig. 2:**
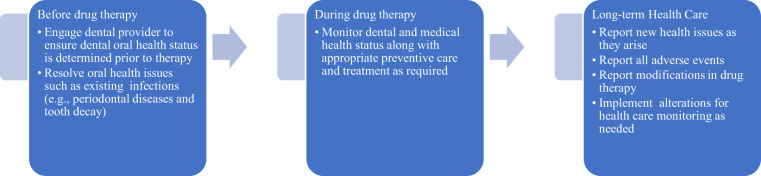
Monitoring of oral health status by health care providers for patients on antiresorptive medications, before, during, and long term

Once on osteoclast-modulating therapy, the American Dental Association (ADA) recommends treatment of patients with active dental or periodontal diseases because failure to do so can lead to complications that require extensive invasive treatment and prolonged care.^35^ A dental practice-based research networks (PBRNs) study shows that dental extractions are associated with ONJ, while other dental conditions and procedures that do not directly injure the bone will not increase the risk of ONJ, thereby supporting the position of the ADA and an increasing number of other societies to provide routine dental care during antiresorptive therapy.^36,37^ In a 2016 Cochrane review of the management of ONJ,^38^ the most recent published large-scale review of management of MRONJ, a combination of prophylaxis, more frequent dental examinations at 3-month intervals, and preventive treatment were found to be more effective than standard care for reducing incidence of ONJ in individuals taking intravenous (IV) BP for advanced cancer. The role of health care providers in prevention and monitoring for MRONJ is summarized in Fig. 2 with steps that begin before drug therapy, during, and long term.

As the optimal duration of BP therapy is unclear, temporary discontinuation of BPs, or drug holiday, has raised considerable debate, especially after long-term use in osteoporosis to reduce risk of fragility fracture. The question of risk of ONJ and atypical femur fracture (fragility fracture) in patients on long-term BPs has prompted the American Society for Bone and Mineral Research (ASBMR) to assemble a task force to weigh in on BP drug holidays. The task force reviewed the risks and benefits of BP through two clinical trials examining BP use for vertebral fracture reduction in osteoporotic patients.^39^ The ASBMR report noted that for women not at high fracture risk after 3–5 years of BP treatment, a drug holiday of 2–3 years could be considered, although a drug holiday would be based on clinical judgment with periodic reassessment. There are no clinical studies identifying an actual decrease in risk of ONJ by stopping BPs, and in fact, a retrospective national database analysis in Korea^40^ has shown a significant number of ONJ cases occurring after BP discontinuation. A 2014 update on MRONJ by the American Association of Oral and Maxillofacial Surgeons (AAOMS)^2^ cautiously recommends a drug holiday of 3 months prior to and 3 months following invasive dental surgery if the patient has been on extended therapy and medical conditions permit the drug holiday. A drug holiday for DNB is not recommended, as bone turnover reverts after 6 months of drug interruption, increasing fracture risk.^41^ The continuing uncertainty about use of drug holidays prompted a close look at the appropriate use of drug therapies for osteoporotic fracture prevention in October 2018, culminating in a systematic review of knowledge gaps and recommendations for new osteoporotic drug research to inform long-term use.^42^ Pretreatment dental care and comprehensive care during BP therapy to eliminate dental sources of infection and avoid subsequent dental extractions remains an important cost-effective recommendation.

Achieving oral health before starting BP therapies targeted at preventing, controlling osteoporosis-related fractures, is a goal that highlights the vital relationship between oral health and systemic health. Managing patients at risk for MRONJ include avoiding trauma to the jaw bones and maintenance of oral hygiene through preventive dental care. While the management for those who have developed MRONJ is beyond the scope of this review, the goal is to limit the progression of the disease, control secondary infections, and limit pain while maintaining the best quality of life for these individuals.

## Risk factors

### Dental risks

Risk factors for MRONJ include oral infections and associated inflammation, e.g., untreated caries, pulp infections, and periodontal disease, as they may lead to extraction of nonsalvageable teeth.^43,44^ The risk for MRONJ is multifactorial but the vast majority of cases include trauma or injury to the jaw bones, particularly dentoalveolar surgery (0.5% risk of ONJ for patients on oral BP; 1.6%–14.8% risk when patients are on IV BP) verified by Dental PBRNs studies.^36^ Under normal conditions, with no history of antiresorptive medications, dental injury or extraction induces woven bone formation as an initial stage of wound healing and requires deposition of collagenous and noncollagenous proteins to promote mineralization. In a mouse model, animals treated with either BP or DNB, tooth extraction sites showed absence of woven bone, impaired bone remodeling, and incomplete wound closure, suggesting a mechanism for onset of ONJ.^45^

Among cancer patients on IV BPs, denture wearers were noted to have at least a twofold greater risk for ONJ, likely resulting from chronic irritation of tissues.^46,47^ Patients and their dentists are encouraged to monitor the dental prosthesis during IV therapy for adequate fit and cleanliness in order to avoid injury to the oral mucosa. In the FREEDOM Extension study following postmenopausal women on DNB for up to 10 years, dentures were also considered an inciting event that was associated with an increased ONJ incidence.^48^

In the 2014 AAOMS Position Paper on ONJ, four stages of ONJ were defined for patients on oral or IV BP therapy including a Stage 0 category. While Stage 0 includes pain, nonspecific clinical symptoms without exposed bone or fistula, and radiographic changes, studies have shown that 50% of patients with Stage 0 will progress to higher stages that include exposed bone or fistulae over time,^49,50^ validating the importance of identifying symptomatic patients prior to disease progression. The staging of ONJ has provided treatment guidelines to prevent progression of disease.

### Medical comorbidities and duration of use associated with increased risks

Most cases of MRONJ are related to IV BP use in cancer patients (approximate incidence of 1% on BP alone), with those who are on concomitant steroids, chemotherapeutic agents, or other antiresorptive or antiangiogenic therapies at greatest risk (with reports of overall incidence upwards of 6% on combination therapies).^51–55^ A few rare cases of ONJ have been reported in cancer patients taking only tyrosine kinase inhibitors without a history of BP or radiotherapy.^56,57^ Three large clinical trials reported up to 16% ONJ incidence in patients taking BP and the antiangiogenic therapy Avastin (IV bevacizumab for colorectal, lung, breast, and renal cancer),^51^ which represented an apparent increase in risk with BP exposure in this population. In addition to bevacizumab, two other nonbone drugs approved by the US Food and Drug Administration (FDA), Sutent (sunitinib malate for gastrointestinal and pancreatic tumors and renal cell carcinoma), and Nexavar (sorafenib for hepatocellular carcinoma), have potential roles in increasing risk for ONJ, most likely by interfering with VEGF signaling.^58^ Disruption of VEGF could compromise the integrity of the vasculature in the jaw, or by causing host defense impairment. ONJ has been reported in patients with no history of antiresorptives or antiangiogenic therapy, but with other medical comorbidities, including systemic infections, rheumatoid arthritis, diabetes, or vascular disease. Greater number of medical comorbidities, rather than one single comorbidity, increased risk of ONJ.^59^

The duration of treatment is a risk factor for the development of MRONJ for both humans and animals. Though several cases of ONJ have been described in association with oral BP for osteopenia and osteoporosis, the risk remains exceedingly low for oral antiresorptive therapy (<0.1%). The risk may increase with >4 years of treatment, but may not outweigh the benefits of osteoporosis therapy to avoid fragility fractures.^60,61^ There may be a risk plateau of ~1% at 2–3 years of exposure to high-dose IV ZOL or DNB in cancer patients,^55,62^ while osteoporosis patients receiving oral BPs may see the greatest risk, 0.21%, after 4 years of oral treatment.^63,64^ The marked contrast in the incidence of ONJ between these treatment options may be related to the difference in the immunological competence and wound-healing ability of cancer and osteoporotic populations, in addition to the dose-associated risk and greater bioavailability with IV formulations. Similar results were seen in rat ONJ models where multiple linear regression showed treatment duration, but not dose, as a significant predictor of overall histopathologic BRONJ prevalence.^65^

While most cases of ONJ occur in patients undergoing treatment for cancer metastasis or osteoporosis, other diseases sharing the same pathways that influence bone homeostasis may also carry a risk for ONJ. Sjögren’s syndrome (SS) is an autoimmune disease in which TGF-B/Smad2 signaling and matrix metalloproteinases are dysregulated, leading to hypofunction of salivary and lacrimal glands.^66–68^ Data from more than 13 000 patients with SS in a nationwide Longitudinal Health Insurance Database from 2000 to 2013 in Taiwan found an increased risk of developing BRONJ following tooth extraction compared with patients without SS (adjusted HR = 7.635, 95% CI 3.126–18.649, *P* < 0.001). The study recognized the additional risk was associated with use of BPs (particularly nitrogen-BPs such as ZOL that influence Smad-dependent signal transduction) and male gender, but not radiotherapy, chemotherapy, or steroids.^69^

### Risks over the lifespan and gender

Children with osteoporosis have been treated with IV BP for many years and a long-term retrospective study of those with osteogenesis imperfecta (OI) has not identified any presenting with ONJ.^70,71^ The lack of these observations suggest differences in aged bone that are risk factors for ONJ. While advanced high-resolution imaging modalities such as quantitative computed tomography provide measures of bone mineral density (BMD), they are not ideal for teasing out differences between comparative osteoporotic samples of varying ages. Fragmentary secondary osteons (an indicator to determine skeletal age in forensic medicine) demonstrate a relative density increase after repeated remodeling over the lifespan and may be a clue to the increased risk of ONJ with age.^72^ Alterations in fibril orientation, collagen crosslinking carbonate substitution, and configuration of other matrix components, contribute to changes in mechanical behavior of bone. There are few clinical tools that are sensitive to age-related changes in matrix composition^73^ and it remains to be seen how macro- and micro-structure of bone influence ONJ incidence.

An early literature review through 2006 of risk factors in patients receiving BP for osteoporosis reported age >60 years and female sex as two of the most common characteristics for those who developed ONJ.^74^ This same finding of females aged 60 years or older was reported in a review of ONJ cases in patients receiving DNB.^75^ It may very well be that osteoporosis, and hence the need for antiresorptive medications, is more common in older postmenopausal women. In a study specifically examining the elderly that included analysis of ZOL as well as DNB-treated patients, those >70 years of age had an increase in MRONJ frequency, with DNB-treated onset occurring as early as 1 year.^76^ This retrospective study, drawing from the Japanese Adverse Drug Event Report database, also noted a higher occurrence in women, accounting for 56% of ZOL and 55% of DNB cases. On the contrary, data from the HORIZON-Yearly Pivotal Fracture Trial indicated ONJ in healthy postmenopausal osteoporotic women on ZOL infusions were low, with only one case observed in the treatment group (out of 3 248 patients).^77^ Population differences in these two studies include a predominantly Japanese cohort versus the latter where only 0.2% Japanese were among mostly white participants. A literature review in 2019 concluded that there is limited scientific evidence for increased risk of ONJ in older individuals.^78^ Although many studies on osteoporosis enroll elderly postmenopausal women who make up a large portion of affected individuals, further studies are needed across the lifespan and with a focus on sex as a biological variable.

### Molecular, cellular, and genetic risk factors

With tools and technologies advancing in genomics, genetic links have been identified and are being explored for conditions affecting the dental, oral, and craniofacial tissues.^79^ Genetic and epigenetic studies have examined whether there are individual risks for developing ONJ in patients taking antiresorptive medications. Table 3 summarizes these other risk factors described here that need to be considered along with the dental and medical factors. A pharmacogenetic study of farnesyl pyrophosphate synthase (FDPS), the enzymatic target of BP, found the A allele frequency of the A/C rs2297480 polymorphism correlated positively with the occurrence of ONJ after 18–24 months of treatment with zoledronate in a Caucasian cohort.^80^ This polymorphism in the FDPS gene could be responsible for germline sensitivity to drug action and might identify patients at higher risk for developing ONJ. Cytochrome p450 CYP2C8 is a main metabolizer of drugs in the body and was discovered through a genome-wide association study (GWAS) to have a single nucleotide polymorphism (SNP) that was significantly associated with a higher risk of ONJ development (odds ratio 12.75) in patients with multiple myeloma undergoing BP therapy.^81^ However, a later study in a prostate cancer cohort found no association between CYP2C8 and ONJ.^82^ Another GWAS study, an exome-wide association analysis, in individuals on IV BP identified two SNPs on chromosome 10 along with two promoter regions of the SIRT1/HERC4 locus associated with BP-induced ONJ.^83^ SIRT1 is a molecule involved in the Wnt signaling pathway and translocates into the nucleus to initiate transcription;^84^ HERC4 is an E3 ubiquitin ligase known to regulate osteoblast (OB) function.^85^ While these few studies suggest genetic risk factors, how they contribute to ONJ is not well understood.

**Table 3:** Candidate and known non-pharmacological risk factors for ONJ: based on animal models and human studies

Risk factors	Source of information (A or H)	Ref.
**Dental factors**		
Oral infections and associated inflammation	A, H	^16,43,44,108,110,114,118,119,123,124,168^
Periodontal disease	A	^44,121,125,166,167^
Untreated caries	H	^44^
Pulp infections	A	^43,44^
Dentoalveolar surgery	A, H	^36^
Trauma or injury	H	^36,114^
Tooth extractions or removal of failed implants	A, H	^45,120,171^
Ill-fitting dentures	H	^46–48^
**Medical factors**		
Systemic infections	H	^59^
Rheumatoid arthritis	H	^59^
Diabetes	H	^59^
Vascular disease	H	^59^
Sjögren’s Syndrome	H	^69^
**Other factors**		
Concentration and duration of antiresorptive drug use, type of antiresorptive drug	A, H	^51–58,60–65,88–90,96^
Gender	H	^74,75^
Age	A, H	^70,71,74–78^
Farnesyl pyrophosphate synthase (FDPS)	H	^80^
Genetic considerations (CYP2C8; SIRT1/HERC4)	H	^79,81–83^
Biodistribution to specific anatomical sites	A	^92,96,98,99,109,115^
Cellular physiology with greater bone turnover	A	^87,96^

*A* animal, *H* human

Biomarkers may provide opportunities for determining risk of developing ONJ, as well as for monitoring risk during antiresorptive therapy. Fourteen bone metabolism and remodeling regulatory microRNAs, which were markedly elevated in patients with ONJ, were tested for their predictive power for ONJ. Three candidate microRNAs, serum miR-21, miR-23a, and miR-145, were used to develop an index for diagnosis. These molecules collectively were found to discriminate between patients receiving antiresorptive therapy without incidence of ONJ versus those developing ONJ. This promising biomarker panel performed better than individual circulating microRNAs, although a subsequent systematic review of all molecules detectable in serum and urine of BP-treated ONJ patients concluded that there are no useful markers to evaluate ONJ risk.^86^ This review noted that of the seven total bone turnover, angiogenesis, and endocrine biomarkers identified (bone alkaline phosphatase, C- and N-terminal telopeptides of type I collagen, deoxypyridinoline, osteocalcin, parathyroid hormone (PTH), and VEGF), the majority of these showed conflicting results. Among the most promising candidates to predict the risk for ONJ may be angiogenesis and endocrine biomarkers, VEGF and PTH, respectively.^87^

## Mechanism of action

### Site of action

Nitrogen containing BPs, such as ALN, ibandronate (IBN), pamidronate (PAM), risedronate, and ZOL, affect osteoclast apoptosis by interfering with the mevalonate pathway for cholesterol biosynthesis. Nonnitrogen containing BPs, such as clodronate, etidronate (ETI), and tiludronate, exert their effects on osteoclasts by competing with adenosine triphosphate (ATP) as a substrate significantly impacting their function to resorb bone. While these effects are not specific to osteoclasts, osteoclasts are exposed to localized concentrations of BPs that are released when bone is resorbed, as BPs are bound very tightly to bone mineral, have an average half-life of 11 years, and exert their pharmacological effect by their target location in a concentration-dependent manner.^88–90^

The predisposition to medication-related osteonecrosis for craniofacial bones, particularly the mandible and maxilla, is unique since osteoclast inhibition and diminished vascularity can be present anywhere in the body. Skeletal site-specific effects of BP on bone remodeling can be distinct, even within the same individual. Craniofacial bones undergo intramembranous ossification and are derived from the neuroectoderm, whereas the peripheral skeleton undergoes both intramembranous and endochondral ossification and are derived from mesoderm. These sites were shown to undergo disparate rates of bone remodeling upon injury in a rat model treated with ZOL, with jaw bone cells being more susceptible to effects of BPs. Using a rat model, researchers noted that ZOL treatment suppressed Wnt-3a expression and decreased the ratio of RANKL to OPG, resulting in limited remodeling at tooth extraction sites versus drill hole-prepared sites in the tibia and ilia. At the cellular level, bone marrow stromal cells (BMSCs) derived from jaw bones exhibited lower proliferative and differentiation capacity and weakened osteogenic and chondrogenic potential compared with BMSC from peripheral bones in these animals.^91^

In addition to a twofold greater incidence in the mandible than in the maxilla, osteonecrosis favors particular sites within human jaw bones and this may be due to local BP accumulation to toxic levels. For example, the necrotic process was found to be more often localized to the posterior mandible compared with the anterior mandible in patients.^92^ Teeth in the posterior quadrants of the dentition are subjected to four- to seven-fold higher biting forces^93^ generated by the mechanics of the temporomandibular joint.^94^ Under such differences in load, the supporting alveolar and maxillary bone structures undergo load-dependent bone remodeling with bone resorption seen under extreme loading.^95^ Preferential binding of labeled ZOL to certain anatomical bone sites was recapitulated in animal models with distinct areas of the posterior mandible (ascending ramus and mandibular molar alveolar bone) demonstrating increased fluorescence labeling.^96^ Similar anatomical differences were also seen in the femur with more labeling in the proximal area of primary spongiosa than distal region, which may explain the increased risk of avascular necrosis of the femoral head. However, the reason for the predilection of MRONJ to the jaw bones and not long bones is unclear. In vitro controls of the fluorescently labeled ZOL exhibited homogenous binding characteristics to standard calcium phosphate discs, in contrast to the heterogeneity in binding seen in vivo, suggesting an influence of biological factors and anatomic variation.

Biodistribution of IV-administered BPs differs markedly based on the protocol and contributes to the risk of ONJ. High-dose infusions for cancer patients and IV administration have different kinetics of adsorption compared with the use of lower oral doses over extended periods of time for patients being treated for osteoporosis. In addition, an oral administration route provides less bioavailable drug due to lower gut absorption.^97^ In rats given a single bolus injection, intense localization to the mandible was seen in the alveolar process of the jaw bone, whereas repeated injections of lower concentrations but at the same cumulative dose resulted in diffuse distribution to bone surfaces.^96^ The high concentration of BP localized at the jaw bone from the bolus injection may contribute to the development of ONJ lesions and explain the higher prevalence in patients treated intravenously.^98^ Hokugo et al. conclude the probability of developing ONJ in the alveolar bone, where bone remodeling is thought to be most active, could be reduced by choosing different administration protocols of BP.^96^

Other considerations of differences in anatomical susceptibility to ONJ may be related to the neuroskeleton. Evidence exists that the mandible periosteum envelope versus the femur envelope are innervated by different sympathetic pathways.^99^ In addition, the protein encoded by *TRPV4*, a calcium-permeable cation channel important in trigeminal pain signaling, is important for vasoregulation and osteoclast differentiation.^100^ The novel finding of prolonged TRPV4 channel openings associated with the pathogenesis of osteonecrosis reinforces the importance of neuroskeletal components in bone disease. Multifunctional molecules such as these need to be studied in the context of their combined mechanisms to understand the interplay of pathways affected in ONJ.

Interestingly, while the effects of BPs on osteoclast function and susceptibility to ONJ may be selective to specific neuroskeletal sites, certain BPs, particularly first-generation drugs such as clodronate, have been used for decades as analgesics, acting as vesicular ATP release blockers in sensory nerves to control neuropathic pain.^101^ The BPs clodronate and ETI block the release of inflammatory cytokines including tumor necrosis factor alpha (TNF-α) and interleukin-6 (IL-6).^102^ This may explain the effectiveness of BPs for bone pain in skeletal dysplasias such as OI and fibrous dysplasia in McCune Albright syndrome.^103,104^

### Delayed wound healing and soft tissue effects of BPs

Wounds in the oral cavity involve the interaction of several soft and hard tissue types. In addition to interference with healing of bone trauma, antiresorptives can compromise proliferation, migration, and differentiation of vascular endothelial cells, delaying vessel remodeling and soft tissue repair in the oral mucosa.^105–107^ Histomorphometric analyses of short-term ALN administration in a tooth extraction wound model in mice revealed sustained inflammation contributing to delayed resorption of damaged bone.^108^ BP-affected osteoclasts were also reported to be found in the connective tissue rather than near the surface of bone.^109^ In mice treated with DNB, palatal bone denudation surgery resulted in similarly suppressed osteoclasts and significant inflammation in the subepithelial palatal soft and hard connective tissue,^110^ delaying wound healing. Osteomucosal healing involving both hard and soft tissues, growth of lymphatic vessels, as well as lymphocytes and cells of the innate immune system may also play important roles in the pathogenesis of ONJ.^111^

Soft tissue toxicity in patients treated with amino-BPs has been reported, most frequently occurring as gastrointestinal, esophageal, or oral ulcerations.^112^ Although clinical evidence for contribution to esophageal cancer is weak, in vitro studies suggest that nitrogen-BPs affect cell growth of stratified squamous epithelia,^113^ and the BPs PAM and ZOL may have direct effects on epithelial cells, impairing soft tissue healing.^114^ The resulting ulcerations occur when oral keratinocytes are not able to proliferate, as they are needed to maintain a barrier to physical, microbial, and chemical agents, and to participate in inflammatory responses to oral infections. In oral mucosal tissue constructs, Lee et al.^114^ showed this inhibition of keratinocyte proliferation occurs through transcriptional downregulation of cyclin A2, which is needed for S-phase cell cycle progression to cell division. Normal human oral fibroblasts, on the other hand, were not affected, suggesting amino-BPs affect specific cell types. This study highlights the importance of soft tissues such as the epithelium, the specific cell type, and consideration of the specific drug in the mechanism of action as there may be a separate route outside of hard tissue pathology that leads to ONJ.

### The immunologic interplay in ONJ

Almost all the drug regimens mentioned here involve a route of systemic administration and hence are systemically bioavailable. BPs, which mimic the natural pyrophosphate structure (except that oxygen is replaced by a carbon to prevent degradation to Pi), readily bind to hydroxyapatite and thus are ideal for skeletal targeting for treatment of bone-related conditions. However, tissues and organs that are not the intended target are exposed to these compounds, and have been demonstrated to be labeled by fluorescent BP compounds.^115^ One such unintended target is the macrophage. In the presence of ZOL, macrophages had elevated TLR-4 expression that altered M1- and M2-macrophage polarization, resulting in activation and production of inflammatory cytokines.^116^ This response could be attenuated in TLR-4^−/−^deficient mice or by a TLR-4 inhibitor, indicating that TLR-4 macrophage polarization participates in the pathogenesis of ONJ.

Tseng et al. further analyzed the effect of BP on osteoclasts and immune cell function in the oral cavity. Their study revealed 27 cytokines and growth factors released from osteoclasts that were found to be different from dendritic cells and M1 macrophages but resembled untreated monocytes and M2 macrophages.^117^ ZOL-treated osteoclasts also activated the function of immune effectors such as natural killer cells and may establish chronic inflammation leading to ONJ pathology. Release of proinflammatory mediators also depended upon the BP used, with ZOL and ALN mediating significant release of interleukin-6, tumor necrosis factor alpha, and IL-1B, whereas ETI did not. IL-6 is a proinflammatory cytokine produced by macrophages, keratinocytes, and endothelial cells, and not surprisingly, salivary levels of IL-6 are increased markedly in patients with advanced stages of ONJ.^118^

A series of reports by Nishimura et al. provided evidence that ONJ disease severity was regulated by the immune system through gamma-delta T (*Tcrd*) cells involved in oral barrier immunity. Compared with wild-type mice treated with ZOL, *Tcrd*-deficient mice exhibited fewer bone-exposed lesions and more pustule/fistula phenotypes. Reintroducing these T cells into ZOL-treated Rag2 immunodeficient mice, which lack oral mucosal inflammation and did not develop ONJ, resulted in reappearance of hyperplastic oral epithelia as expected.^119^ Prolonged oral inflammation and gamma-delta T cells appear necessary for this pathological condition. Similarly, depletion of Ly6G^+^/Gr1^+^ myeloid cells in the gingival oral barrier tissue attenuated ONJ-like lesion development and was specific to the extraction site, as bone marrow myeloid cells were unaffected.^120^ Taken together, aberrant oral barrier immunity can significantly disrupt oral wound healing providing a basis for ONJ pathogenesis.

### Microbiota and inflammation

Periodontal disease, periapical infection, oral restorations (e.g., dentures, implants, and crowns), and any wounds that breach the oral mucosa barrier trigger multiple reactions. Experimentally induced periodontitis and periapical disease are common models to study the effect of localized inflammation on ONJ development. Animal models have demonstrated periodontal disease without bone injury as a risk factor for developing ONJ. However, periodontal disease alone without tooth extraction or dental implant placement does not reach statistical significance.^121^

In the mouth, exposed bone is heavily colonized by oral bacteria with Actinomyces being the most frequently reported bacteria identified in BRONJ lesions.^122^ Through 16S rRNA sequencing, a unique set of species and phylotypes have been found exclusively in ONJ that are not found in individuals who have a history of BP without ONJ.^123^ This finding alone does not explain whether ONJ is triggered by infection or if exposed necrotic bone is colonized by this specific biofilm. However, it is clear that there are significant changes in host genes regulating immune function in ONJ patients as a result of their microbiome. These changes include downregulation of key genes and modulators required to mount antibacterial defense, resulting in a deficient innate immune response allowing colonization and biofilm formation in ONJ tissues. Removal of plaque and debris from exposed bone with chlorhexidine has been reported to have a beneficial effect, decreasing the influence of microbiota and time to disease resolution.^124^

Medications shown to affect specific microbes could play a role in modulation of the host immune system, and hence bone physiology. BPs themselves may participate in this path through the microbiome. In a series of in vitro experiments testing the effect of BP on oral and non-oral bacterial strains, the BPs IBN, PAM, and ZOL were found to inhibit a range of bacterial species, including *A. actinomycetemcomitans*, *C. ochracea*, and *C. rectus*,^125^ common periodontal pathogens. Perturbation of the commensal microbiota in the gut with agents such as BPs and antibiotics could represent a consideration in the influence of immune crosstalk^126,127^ and hence dysbiosis contributing to ONJ pathology. Intervening at the level of microbe-host immune interactions supports the approach of managing infection with antibiotics and antimicrobials to aid resolution of ONJ.^128^

While bacteria and their byproducts are the primary instigating agents, the host releases many proinflammatory cytokines, chemokines, and reactive oxygen species that contribute to oxidative stress causing cell damage, host tissue destruction, and poor wound healing.^129^ For example, epithelial cells adjacent to ONJ lesions upregulate expression of the proinflammatory cytokine IL-36a. IL-36a signals through the NF-KB and Erk pathway preventing nuclear translocation of the Smad2/3 complex, thus inhibiting collagen synthesis in gingival cells.^130^ The etiological role of IL-36a in ONJ was confirmed in a mouse model where knockdown of an IL-36 receptor subunit ameliorated the condition.

### Bone biology associated with ONJ

Various cell types have been reported to contribute to initiation of MRONJ. Biochemical studies have shown the mechanism of action of BP in osteoclasts is through inhibition of the mevalonate pathway and as a cytotoxic ATP analog.^131^ Pathological studies using imaging of the alveolar bone revealed osteoclasts filled with reservoirs of ZOL detached from the surface of the bone and exhibited a rounded and degenerated phenotype, loss of polarity, and pyknosis.^132^ This aberrant localization of BP-affected osteoclasts in gingival connective tissues rather than on bone surfaces was verified in situ using labeled ZOL.^109^

Compared to osteoclasts, the scientific literature regarding the effect of BPs on OBs and osteocytes (OY) is less conclusive. Early evidence showed OB retains its function and is not adversely affected by BPs. For example, Jeong et al. reported that BMP2 stimulation of cultured OBs previously treated with ALN resulted in bone formation.^133^ Further studies demonstrated BPs directly prevent OB as well as OY apoptosis independent from the effect of BPs on OC and strictly work through opening of connexin-43 hemichannels, thereby activating prosurvival signals.^134^ This effect was verified in primary murine OB isolated from calvaria^135^ but seems limited to nonnitrogen BP as amino-BP PAM and ALN were later reported to cause OB apoptosis and suppressed OB differentiation, respectively.^136,137^ ALN was shown to be taken up by OB in sufficient amounts to inhibit protein prenylation, causing retarded OB growth and eventual OB apoptosis.^138^ The conflicting apoptotic and antiapoptotic effects could be explained by the different BPs studied and concentrations used,^139^ with concentrations above 10^−5^ mol · L^−1^ mostly being inhibitory. This dual nature was likewise seen in pre-OB where micromolar concentrations of ZOL and ALN decreased pre-OB mineralization and were cytotoxic, whereas lower doses increased proinflammatory mediators TNFa and IL-1B, increased inhibitors of osteoblastogenesis, and decreased expression of collagen and osteopontin.^140^ Mounting evidence suggests that cells of the OB lineage are affected directly by BP in a dose-dependent manner that contributes to the development of ONJ, thus warranting attention.

OY are considered mature OB embedded within the bone and continue to be key regulators of bone homeostasis. As mentioned above, negative effects on cells of the osteogenic lineage by BP could result from exposure to high concentrations over an extended period of time. Remodeling could be negatively affected resulting in an imbalance for not just bone formation^141^ but bone healing. The role of osteocytes in the context of cell death has been explored through glucocorticoid-induced osteonecrosis. Glucocorticoids rapidly and strongly repressed MMP13, a key perilacunar remodeling (PLR) enzyme, in both trabecular and cortical bone of the mandible, and induced OY apoptosis in mice.^142^ The result is reduced bone volume, trabecular thickness, BMD, and flexural strength^143^ of the mandible and a disrupted osteocyte-perilacunar-canalicular network. Impairment of the canalicular network in mice has been seen with oxidative stress, which has a particular effect on osteocytes and suppresses bone turnover.^144^ Regions of necrosis in alveolar bone with nonviable osteocytes are present in BP treatment.^145^ Without osteocytes to drive PLR to maintain the bone matrix and hence bone quality,^146^ all the hallmarks of osteonecrotic lesions occur.^147^ Many years before the increase in cases of BP-induced ONJ appeared in the literature, Weinstein et al. introduced the concept of accumulated apoptotic osteocytes contributing to osteonecrosis.^148^

### Mechanisms informing additional therapies

Data from studies focused on evaluating the outcome of several promising regenerative therapies in conjunction with combined chemotherapeutic and BP therapy in ONJ models have contributed to understanding of tooth extraction site healing. Systemic transplantation of stromal vascular fraction cells into the tail vein of mice, and mesenchymal stromal cells sheet transplantation onto the mandibular bone in beagles, was found to ameliorate the ONJ-like lesions in tooth extraction sites.^149,150^ This therapy improved both osseous and soft tissue healing, most likely by increasing the number of blood vessels, and reducing TRAP^+^ mononuclear cells and nonattached osteoclasts from the bone surface around the extraction sites. A 2016 comprehensive review discusses the importance of bone turnover in pathogenesis of ONJ and canvases the studies on use of mesenchymal stem cells (MSC) and other therapies.^151^ Overall, MSC grafts were beneficial in treating ONJ by counteracting many of the mechanisms mentioned in the preceding sections through stimulating OBs, bone formation and bone remodeling, and decreasing inflammation.

New discoveries of therapies that decrease necrotic bone and resolve osteonecrosis may assist in defining the factors or cells promoting MRONJ. In a January 2019 study using locally administered polydeoxyribonucleotide (PDRN) from salmon sperm, osteonecrosis in a BP-induced molar extraction rat model was resolved.^152^ PDRN treatment lowered necrotic bone and increased the number of blood vessels, and also led to recovery of osteoclast function. PDRN is a stimulator of VEGF production and has been shown to be safe enough to enter clinical trials for diabetic wound healing.^153^

In experimental imaging studies displacing existing BP from bound sites in tissues, Howie et al. demonstrated that systemically delivered chelating agents can remove PAM from all bone surfaces in rats, and of the overall signal observed the maximal reduction occurred in alveolar bone and femur.^154^ Topical local chelation by agents such as ethylenediaminetetraacetic acid was equally efficacious at rescuing osteoclast function and offers a potential therapy to prevent full lesion development around the tooth extraction socket.^155^ Alternative strategies to reversing BP treatment have been to outcompete existing previously administered BP with locally applied weaker BP compounds that have fewer side effects.^156^ Both of these effective methods may be used to potentially prevent ONJ in patients who have been exposed to this subset of antiresorptives.

In another tooth extraction model, limited necrotic lesions in rats receiving ALN and DEX were rescued by PTH administration, which promoted overall tooth socket healing by increasing bone fill and connective tissue maturation.^157,158^ To date, recombinant human PTH (rPTH, teriparatide), a hormone known to affect both anabolic and catabolic functions of mineralized tissues, is the only FDA-approved anabolic agent for the treatment of osteoporosis. Off-label use of rPTH in individuals with ONJ was reported to resolve ONJ^159–161^ though the mechanism of bone healing is unclear.

## Animal models for study of ONJ

Models mimicking the clinical presentation of MRONJ are essential for study of the pathophysiology of this condition and to discover pathways for prevention, treatment, and monitoring outcomes. Animal models that reflect various risk factors, including inflammation (as seen in periodontal disease) and osteoporosis, both of which affect females differently than males, have been highly sought. In a 2018 survey of 139 studies, Holtmann et al. reported 87, 46, and 6 conducted in vivo, in vitro, and both in vivo and in vitro experiments, respectively,^4^ with rats, mice, dogs, and minipigs, the dominant and preferred animal models. Among these, rodent models constituted over 86% of studies and are most common for obvious cost and efficiency reasons. Highlighted below are some examples of how these models were used to further define mechanisms associated with ONJ and is tabulated in a separate column in Table 3.

### Rodent models

The swamp rice rat or rice rat, *Oryzomys palustris*, is not a new model, but has seen increased utility in ONJ research. The finding of spontaneous periodontal disease in the rice rat in the 1950s was followed up with extensive characterization of the cause, its comparison to human disease, and its applicability.^162,163^ The advantages of this model were clear as periodontal disease could be noted as early as 16 days of age, and destructive pathological changes in the bone within 90–100 days, much faster than the years it takes to develop most types of periodontal disease in humans.^164^ Since its anatomical and histopathological findings were known to be linked to diet, further characterization of diet as well as the type of chow revealed food impaction was a common cause.^165^ In a study with 230 ZOL-treated rice rats with localized periodontitis, it was shown that ZOL increased the prevalence of ONJ in a dose-dependent manner. This rice rat model was valuable in relating periodontitis to spontaneous ONJ. The high degree of susceptibility to periodontal disease allowed researchers to test the hypothesis that a localized inflammatory condition in the oral cavity such as periodontitis, and not merely a traumatic event such as tooth extraction, was an important risk factor for ONJ.^65,166^ This supports the clinical observation that not all clinical cases of ONJ result from tooth extraction, as discussed previously.

The Sprague-Dawley rat is another model researchers turned to in the search for clinically relevant models of ONJ. Since it was known that periodontitis is associated with ONJ, an aggressive periodontitis disease model was induced by placing a ligature around the crown of the rat molar. Under administration of zoledronic acid, rats with ligatures developed osteonecrosis that was visualized by microCT and confirmed by histology. The resulting osteonecrosis was similar to ONJ in patients undergoing BP treatment, complete with bone sequestrationm, and periosteal alveolar bone formation.^167^

Several C57BL/6J mouse models were developed hinging on the importance of an inflammatory component in ONJ. Periapical disease was induced by pulpal exposure of mandibular molars in mice, which had been injected with high-dose ZOL. At 8 weeks, radiographic and histologic analysis revealed features resembling clinical ONJ with osteonecrosis, but only a third of these animals developed exposed bone.^168^ Another mouse model that did not involve an experimental surgical intervention, was based on reports of naturally occurring maxillofacial abscesses with significant osteolysis.^16^ Treating these mice with RANK ligand inhibitors, Rank-Fc or OPG-Fc, or ZOL, resulted in ONJ-like lesions at sites of maxillary periradicular infection, supporting the role of osteoclast inhibition and inflammation in ONJ pathogenesis.

### Larger animal models

In a review on the types of in vivo models used to study MRONJ, Holtmann et al. concluded that the minipig was the most suitable animal model because ONJ was reliably induced and the pig’s oral bones and teeth align with human bones and teeth physiology.^4^ Despite this realization, only 3.4% of publications from 2007 to 2017 used pigs. Newer models are being developed such as sheep, which reproducibly demonstrate spontaneous and ZOL-induced ONJ.^169^

Early in the 2000s, efforts to study ONJ in a variety of dog models were begun but have been stymied by lack of consistent exposure of jaw bones after BP administration, even after 3 years of treatment.^170^ BP with corticosteroid therapy generally predisposes to the occurrence of ONJ-like lesions after tooth extraction and has been reported in cats;^171^ however, in beagle dogs, even with high doses of zoledronic acid combined with dexamethasone, exposed bone following dental extraction was absent.^172^ Matrix necrosis was not noted, and all extraction sites healed without incident.

## Conclusion

MRONJ is a rare but potentially devastating side effect of antiresorptive therapy. The unique physiology of craniofacial bones appears to contribute to the increased concentration of BP in this location versus other skeletal tissues and is dependent upon administration route and dosing. In vitro experiments at the cellular level complement these clinical observations and have revealed part of the mechanism. The etiology is complex and remains unclear, and may involve the immune, nervous, skeletal, and vascular systems, coupled with one’s microbiome.

The risk for MRONJ is multifactorial and the vast majority of cases include trauma or injury to the jaw bones, particularly dentoalveolar surgery. Oral infection and inflammation and medical comorbidities are significant risk factors for ONJ and are key contributors to initiation and progression of the disease. Through dental evaluations and management of the patient by the health care provider team before drug therapy, during, and long term, the risk of developing ONJ can be reduced significantly thereby increasing patient compliance and improving clinical outcomes.
